# Experimental Study on Durability and Bond Properties of GFRP Resin Bolts

**DOI:** 10.3390/ma17122814

**Published:** 2024-06-09

**Authors:** Mingan Lin, Fuming Zhang, Wei Wang

**Affiliations:** 1 School of Civil Engineering, Southwest Jiaotong University, Chengdu 610031, China; change21c@163.com; 2 Institute of Defense Engineering, Academy of Military Sciences (AMS), People’s Liberation Army (PLA), Beijing 100036, China; dzj0379@163.com; 3 Key Laboratory of Impact and Safety Engineering, Ningbo University, Ministry of Education, Ningbo 315211, China

**Keywords:** GFRP, anchor bolt, durability, seawater, pullout test, bond performance

## Abstract

Glass fiber-reinforced polymer (GFRP) anchor bolts are a new type of high-performance nonmetallic anchor with significantly higher tensile strength, a lighter weight, better corrosion resistance, and a lower cost than steel bars. Therefore, exploring the durability and bonding performance of GFRP anchor systems is of great importance for the structural design of protective engineering, especially in coastal environments. However, insufficient research has been conducted on the durability of GFRP resin bolts in seawater conditions, with no universal standard on the pullout testing of GFRP bolts. To study the durability and bonding performance of GFRP resin bolts, durability experiments were conducted in this work using artificial seawater, and the pullout tests were conducted using a large-scale concrete platform with different compressive strengths (21.2, 40.8, and 61.3 MPa). The results of the durability experiments indicated that the strength variations of the GFRP rods and epoxy resin materials in artificial seawater environments were less than 5%. Subsequently, indoor pullout tests using steel tubes filled with epoxy resin were conducted, and the test results indicated a critical anchor length value. Pullout tests of the GFRP resin bolts embedded in large-scale concrete blocks were also conducted with different strengths. According to the test results, all GFRP resin bolts embedded in the three concrete blocks with different compressive strengths exhibited rod fracture failure. The failure mode was not controlled via the compressive strength of the concrete blocks due to the high bonding strength between the resin and the rod, as well as between the resin and the concrete. Therefore, this GFRP resin anchor system could fully utilize the tensile strength of GFRP rods. This research offers significant practical value in verifying the safety and reliability of GFRP resin bolts in corrosive marine service environments, and it contributes to the application and development of GFRP materials in the engineering field, serving as a valuable reference for the structural design and further study of GFRP bolts.

## 1. Introduction

Anchor bolts are widely used in tunnel, slope, foundation pit, and anti-floating engineering, as they can effectively improve the strength and stability of the rock/soil [1]. Steel anchor rods accelerate deterioration under various corrosive service conditions. Thus, due to their non-corrosive, high strength, and lightweight properties, fiber-reinforced polymer (FRP) bars have gained popularity in the field of geotechnical anchoring engineering as substitutes for steel bars in harsh environments such as seawater. As a type of non-metal material, glass fiber-reinforced polymer (GFRP) composites serve as promising composites and as an alternative to steel bars in ocean protective structures due to their excellent corrosion resistance. The durability and bonding properties of GFRP composites are considered important factors in the service performance of GFRP anchor bolts. Therefore, it is of great significance to explore the durability and bonding performance of GFRP bolts to fully utilize their tensile capacity.

Understanding the degradation of GFRP composites in corrosion conditions is critical for improving the long-term performance of GFRP materials and promoting engineering applications. Accelerated testing methods are commonly used for the corrosion testing of GFRP materials, which can be classified as heating methods and high-concentration methods to simulate the corrosive environment found in nature. The corrosion media for FRP materials in nature mainly consist of groundwater, contaminated soil, acid rain, concrete, and seawater. Many studies have been conducted to investigate the corrosion resistance of GFRP materials. Specifically, the effects of various corrosion media, freezing–thawing, and temperature [2,3] on the durability of GFRP materials have been reported. Research conducted by Zhang et al. [2] showed that GFRP rods exhibit excellent resistance to acid and salt corrosion but have relatively poor resistance to alkali environments, and the strength and stiffness of GFRP rods showed minimal changes after undergoing 300 freeze–thaw cycles. Kajorncheappunngam et al. [3] investigated the durability of glass fiber-reinforced resin in four different corrosive media (pure water, a 30 g/100 mL NaCl solution, a 5 mol/L NaOH solution, and a 1 mol/L hydrochloric acid) after storage at 60 °C for a certain period of time. Many studies were conducted on the short-term performance of FRP reinforcement structures. However, investigations into their long-term performance have remained inadequate. The study of FRP reinforcement is still in a preliminary stage, with a limited exploration into durability concerns in ocean environments [4,5,6,7]. More work remains to be done in order to provide a better understanding of the durability and long-term performance of GFRP bars. In this study, we presented the influence of seawater on the physical and mechanical properties of GFRP composites using accelerated testing at an ambient temperature.

Many factors can affect the bonding strength of GFRP anchor bolts, such as the bar diameter [8,9,10], anchoring agent [11,12,13], and matrix concrete strength [10,14,15]. The bonding strength between the GFRP bar and concrete will differ from the bonding performance of GFRP ground bolts, and the actual state of geotechnical anchoring engineering would be inconsistent without considering the influence of grouting on anchoring performance in bonding strength tests between a GFRP bar and concrete. Existing studies have primarily centered on concrete structures [16,17], emphasizing the analysis of bonding strength at interfaces with concrete [18] or mortar, with a limited exploration of other geological materials such as sandy soil, clay soil, and various weathered rock masses [19]. Liu et al. [20] conducted experimental studies to investigate the factors influencing the anchoring performance of fully threaded GFRP mortal bolts, using concrete with a strength grade of C60 to simulate rock mass. Huang et al. [21] conducted in situ destructive testing of GFRP rock bolt structures to investigate the influence of rock weathering on the bonding strength of GFRP rock bolts and the structural failure mechanism. The bearing capacity of GFRP bolts and the bond strength in cement mortar were also discussed in the literature [22,23] and compared to steel bolts. GFRP and steel anchors were installed in mass concrete blocks and field rock blocks. The test results showed that the bonding strength of GFRP bolts was close to that of steel bolts. However, research on the performance of GFRP rock bolts in marine environments is notably deficient. No standards currently exist for the test methods and test specimens, with a lack of significant valuable experimental data to support research on bond properties. In addition, conventional anchor agents are mostly cement-based materials, although epoxy resin is recommended and used as coatings for marine applications, more studies on the durability and bond performance of a GFRP resin anchoring system in corrosive environments are still required.

In this paper, we present the durability results of an experimental study in simulated seawater conditions and the bonding performance of GFRP resin bolts embedded in a large-scale concrete matrix with different compressive strengths of 21.2, 40.8, and 61.3 MPa used to simulate rock mass with low, medium, and high strength.

## 2. Corrosion Testing

### 2.1. Preparation of the Corrosive Solution

Seawater contains approximately 3% salt content, with elevated concentrations of Cl^−^, Mg^2+^, and SO_4_^2−^ ions that exhibit strong corrosive properties. To simulate the seawater environment, a corrosive solution was prepared using salt, acid, and water. Industrial-grade salt was used due to its salinity, with 99% industrial sulfuric acid serving as the acidic component, and potable water was added. The main ingredient of industrial-grade salt was NaCl. The solution was accurately measured to contain ion concentrations 5 to 10 times higher than those found in seawater. In this experiment, the accelerated testing method was used at a standard temperature. The precise combination of water, salt, and acid by weight is shown in Table 1.

**Figure 1:**
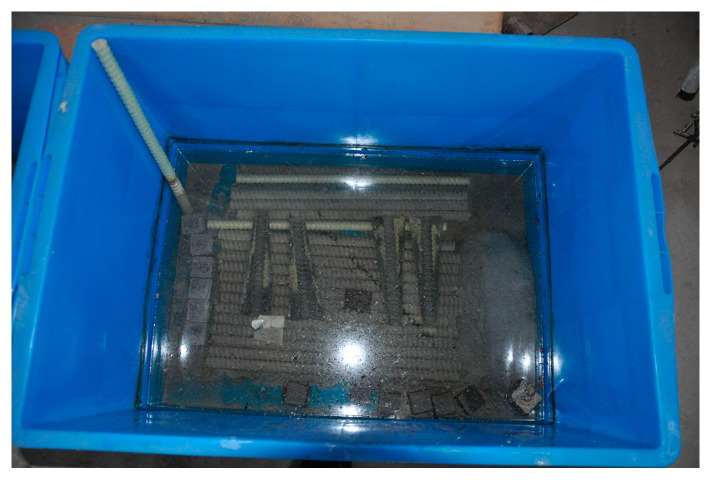
A typical corrosion pool.

A typical corrosion pool used in this work is shown in Figure 1. The corrosion box was fabricated from polypropylene, with a size of 800 mm × 600 mm × 500 mm (length × width × height). In this experiment, six corrosion boxes were used.

### 2.2. Materials and Methods

The GFRP rods were pultruded from E-glass fibers and a Bisphenol A epoxy resin matrix, produced by Nanjing Research Institute of China Coal Research Institute. The fiber ratio was about 65%. The relevant parameters provided by the manufacturer of the GFRP rods used in the experiments are shown in Table 2. Specimens were cut to a length of 50 cm and immersed in the corrosion chamber (Figure 1).

Due to the strong corrosive environment in marine protection engineering, traditional cement-based anchoring agent materials typically exhibit poor durability. To fully exploit the strength advantages inherent to GFRP rods and allow the anchoring system to withstand higher tensile forces, a modified epoxy resin anchoring adhesive featuring corrosion resistance, high strength, good injectability, and simple operation was employed in this experiment. The main technical specifications of the modified epoxy resin materials, produced by Guangzhou Chemical Grouting Co., Ltd. (Guangzhou, China), Chinese Academy of Sciences, are listed in Table 3.

The anchoring agent material was composed of modified epoxy resin and a water-based curing agent with a 1:0.1 ratio by weight. The dimensions of the anchoring adhesive specimens as poured were 4 cm × 4 cm × 5 cm (length × width × height), and the specimens were cured under ambient natural conditions. The compressive strength of the specimens was tested using a WAW-600 computer-controlled electro-hydraulic servo universal testing machine (MTS), manufactured by Shenzhen SUNS Technology Stock Co. Ltd. (Shenzhen, China), with three specimens per group. The compressive strength test results after 3, 7, 14, and 28 days were 19.95, 73.61, 85.68, and 114.55 MPa, respectively.

All experiments in this study were conducted in natural environmental conditions. Twelve GFRP rods were immersed in the corrosion solution for a testing period of 150 days to observe changes in their physical and mechanical properties, such as weight and tensile strength. Additionally, 15 specimens consisting of modified epoxy resin with a water-based curing agent were placed in each of the five corrosion solutions (excluding water) and subjected to immersion testing for 126 days. Immersion testing was conducted in five stages (7, 31, 62, 90, and 126 days), with three specimens tested at each stage to evaluate changes in weight and compressive strength. The tensile strength of the glass fiber rods were tested using a WAW-600 universal testing machine (MTS), with a load speed of 5 mm/min, and the compressive strength of the anchoring adhesive was tested using a WAW-600 universal testing machine (MTS), with a load rate of 1 MPa per second. A total of 87 specimens was immersed in the corrosion solution, comprising of 12 GFRP rods and 75 modified epoxy resin anchoring adhesive specimens, as shown in Table 4 below.

### 2.3. Results and Discussion

**Figure 2:**
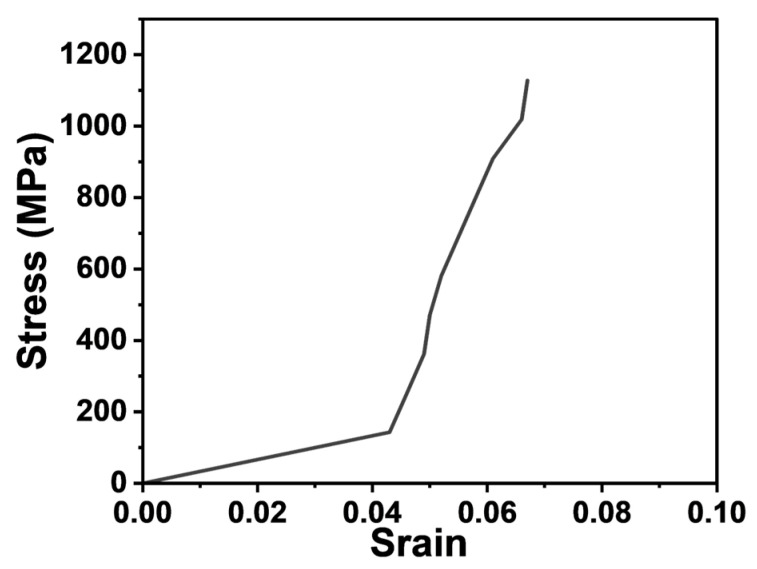
GFRP bar stress–strain curve in tension.

A hollow jack and an electrical-driven oil pump were used for step-wise loading until the failure of the GFRP anchoring bar, with each step at 2 MPa, over the range of 3 to 23 MPa. During the test process, the GFRP bar displacement at each loading step was measured and recorded. According to the test results, the representative stress–strain curve was shown in Figure 2, with a straight-line segment at the starting section, and then it behaved as a steep straight line with slight fluctuations, which were mainly caused by the test process, measurement errors, and the inconsistent deformation of the internal fiber filaments of the GFRP bar during tension. The ultimate tensile strength of the GFRP rods before corrosion was 1130 MPa, with an elastic modulus of 4.4 × 10^4^ MPa and an elongation of 2.14% (Figure 2). After corrosion in various corrosive solutions for 150 days, minimal changes were observed in the tensile strength, elastic modulus, and elongation. In addition, the failure mode remained brittle fracture, which was consistent with the pre-corrosion condition. The experimental results are summarized in Table 5.

The performance of the epoxy resin anchoring adhesive specimens before and after corrosion was evaluated (Text S1 and S2 in the Supporting Information). According to the test results (Text S1 and Table S1, Figure S1 in Supporting Information S1), we observed that, regardless of the corrosive solution, the weight of the epoxy resin material experienced a minimal change with an increasing corrosion time. Nevertheless, the change in compressive strength is slightly significant, demonstrating a substantial linear increase with the corrosion time (Text S2 and Figure S2 in Supporting Information S2). The main reason for the increase in compressive strength may be the maturation after curing, and the presence of corrosive liquid did not seriously damage the strength of the material. In general, the weight and strength variations of the epoxy resin materials in harsh corrosive environments were less than 5%. Hence, this material is considered reasonably suitable for grouting materials in severe environments.

## 3. Pullout Test

### 3.1. Test Design

Currently, no unified standard methods exist for conducting pullout tests on FRP anchor rods. In indoor pullout tests, a typical setup involves the use of grouted steel tubes [24,25], while for field tests, small-scale concrete blocks simulating rock masses are often employed. In this study, indoor pullout tests were conducted using steel tubes filled with epoxy resin anchoring adhesives. For the field tests, a large-scale concrete platform simulating rock mass was utilized, with three different concrete compressive strengths: 21.2, 40.8, and 61.3 MPa.

### 3.2. Materials and Methods

GFRP rods with a diameter of 22 mm and full threads were selected for the experiment, with the relevant parameters detailed in Table 2. The anchoring agent consisted of an epoxy resin material, and its parameters are outlined in Table 3.

**Figure 3:**
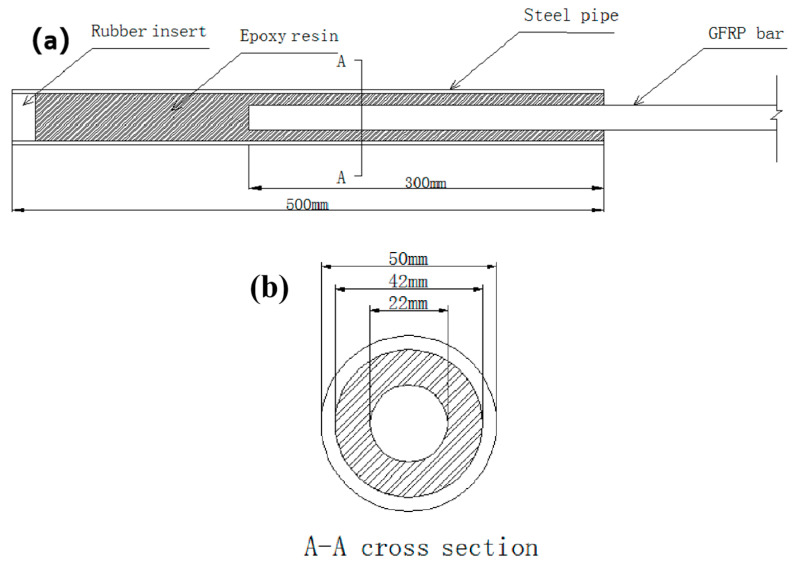
Sketch of steel tube: (**a**) sketch of steel pipe when GFRP rod was inserted at depth of 300 mm and (**b**) anchor cross section.

**Figure 4:**
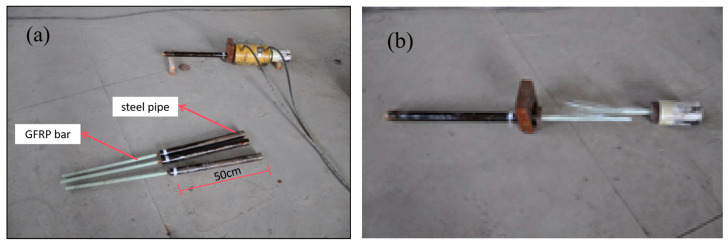
Test specimens: (**a**) before the pullout test and (**b**) after the pullout test.

To determine the critical anchor length, the anchored rods were pulled out from the steel tubes filled with epoxy resin material. For this purpose, 45# steel [26] tubes were selected with a length of 50 cm, a diameter of 50 mm, a wall thickness of 4 mm, and an internally roughened surface (see Figure 3 and Figure 4). A 1-m-long φ22 GFRP rod was then cut and inserted into the steel tubes at depths of 10, 15, 20, and 30 cm. After 7 days, tension tests were conducted using a hydraulic jack.

**Figure 5:**
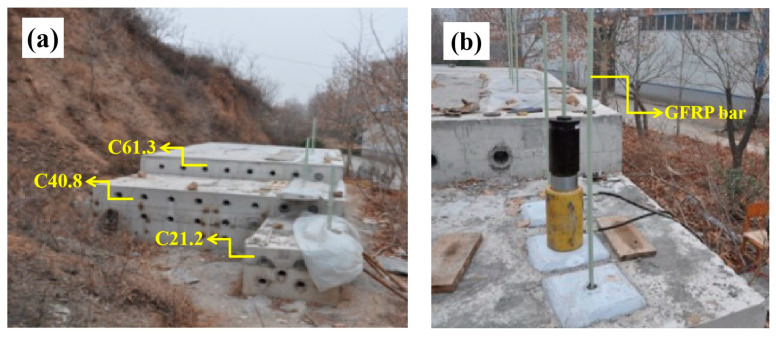
Simulated rock mass test platform: (**a**) concrete platform and (**b**) anchor test.

**Figure 6:**
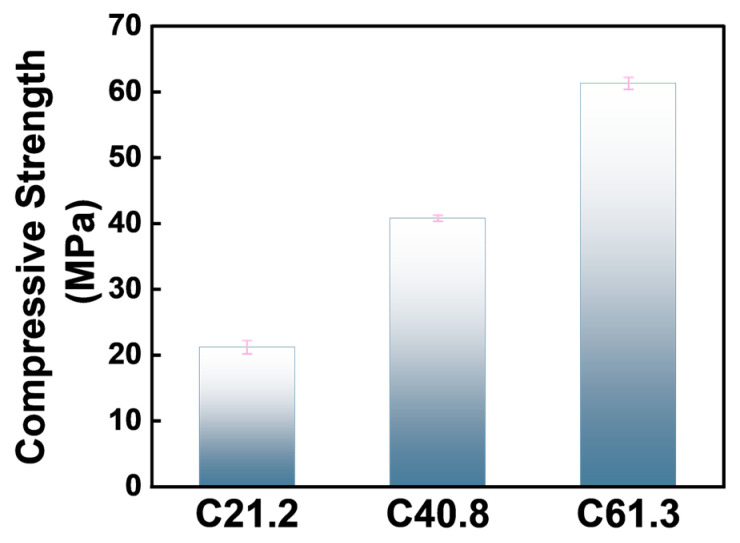
Compressive strengths of the simulated rock mass concrete.

Three types of large-scale concrete blocks with low, medium, and high compressive strengths were chosen to simulate the rock masses for the test platform (Figure 5), with strengths of 21.2, 40.8, and 61.3 MPa. The compressive strength was determined by testing the 150 mm concrete cube specimens cured under standard conditions for 28 days, according to the GB/T 50107 standard [27], as shown in Figure 6 and Table 6. In these concrete platforms, the holes were 30cm deep and drilled with a diameter of 42mm, for a total of 7 holes. These holes were then filled with epoxy resin anchoring adhesive. A 1-m-long GFRP rod with a diameter of 22 mm was cut and inserted into each of the 7 holes at a depth of 30 cm. The hole openings were then sealed with quick-setting cement mortar, and tension tests were performed after 7 days using a hydraulic jack.

### 3.3. Experiment Results and Analysis

Table 7 presents the results of the pullout tests for the four GFRP anchor rods, revealing two types of failure modes, namely slip failure (rod–resin interface slip) and strength failure (rod fracture).

The average bonding strength at the anchoring interface, encompassing the first interface (the inner layer interface, i.e., between the rod and the anchoring adhesive) and the second interface (the outer layer interface, i.e., between the anchoring adhesive and the concrete), was calculated during the pullout process using Equation (1) as follows [28]:

(1) $$\left.\begin{array}{l} \tau_{1}=\frac{F_{\max}}{\pi dL_{a}}\\ \tau_{2}=\frac{F_{\max}}{\pi DL_{a}} \end{array}\right\}$$

where *F*_max_ is the pullout force (kN) when the anchor system failed, $\tau_{1}$ and $\tau_{2}$ denote the average shear stress at the first and second interfaces (MPa), respectively, *d* and *D* represent the diameter of the anchor rod and the diameter of the drilled hole, respectively, where *d* = 22 mm, *D* = 42 mm, and *L_a_* denotes for the anchorage length (mm).

**Figure 7:**
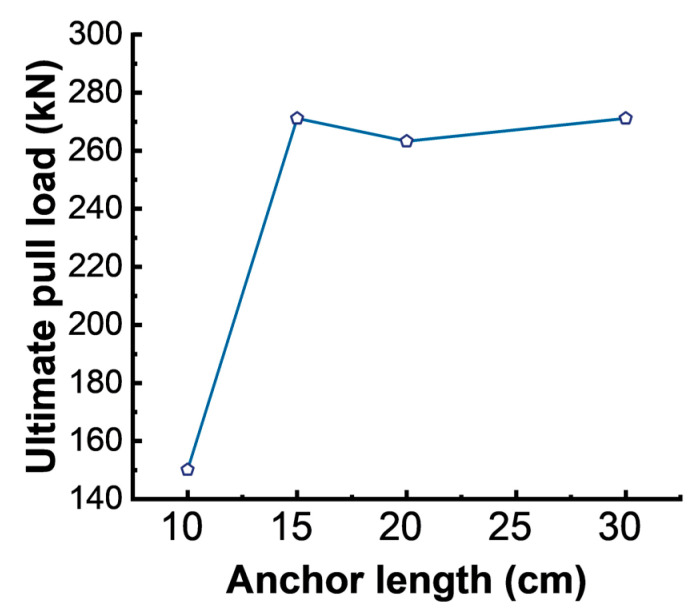
Ultimate pull loads of the GFRP bolts with different anchor lengths.

The failure modes of anchor rods generally include rod body failure, shear failure between the rod body and the anchoring material, and shear failure between the anchoring material and the surrounding rock. The data in Table 7 were plotted into a graph, as shown in Figure 7. As shown in Table 7 and Figure 7, under unchanged conditions, controlling a single factor by merely increasing the anchorage length of the anchor rod could not indefinitely increase the ultimate pullout force of the anchorage system. A critical value typically exists for the anchorage length of GFRP anchor systems, which has been confirmed via existing technical studies [29]. When the anchorage length was less than this critical value, the result of rod pullout would involve either shear failure between the rod and the anchoring material, shear failure between the anchoring material and the concrete block, or a combination of both. The pullout capacity would depend on the average shear strength of the interface, with this anchorage length value referred to as the critical anchorage length. When the bond length exceeded this value, a rod fracture occurred, and the pullout capacity of the anchor system depended on the tensile strength of the rod itself. Rod–resin slip and rod fracture failure were observed in this experiment primarily due to the threaded surface of the GFRP rod and the threaded inner wall of the steel pipe, with both exhibiting relatively high shear strength. Hence, the failure predominantly occurred in the form of shear failure within the anchoring material and rod fracture, which aligned well with practical engineering scenarios.

According to the experimental results shown in Table 7, the critical anchorage length in this experiment was determined as 15 cm. By substituting this value into Equation (1), τ=150.13×1000÷(22×3.14×150)=14.5 MPa, we calculated that the average shear strength reached 14.5 MPa.

**Figure 8:**
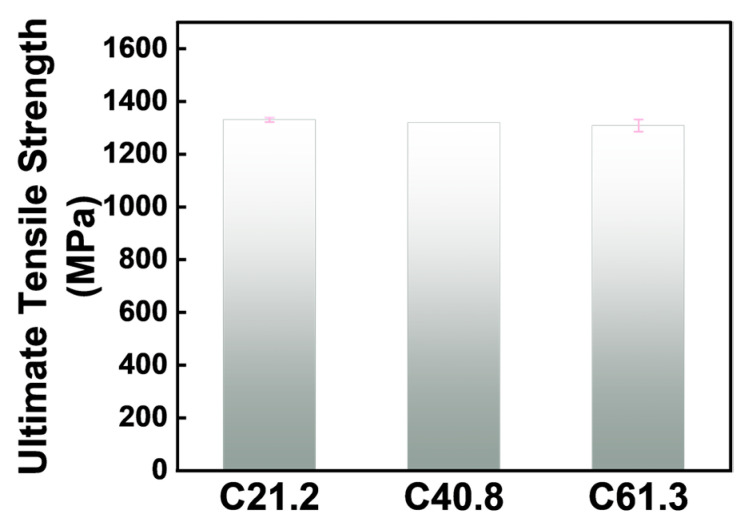
Pullout test results of the GFRP bolts in concrete with different strengths.

The pullout test results of the 7 GFRP anchor rods in the low- (21.2 MPa), medium- (40.8 MPa), and high- (61.3 MPa) strength simulated rock mass concrete samples are presented in Figure 8 and Table 8.

**Figure 9:**
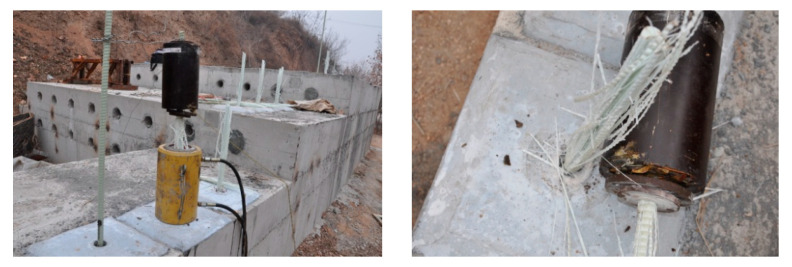
Typical failure state of the GFRP anchor bolts.

All seven GFRP anchor rods exhibited mid-section fractures, with a distinctive “lantern-shaped” rupture pattern indicative of brittle failure, devoid of any apparent precursory signs. The post-experiment failure state is shown in Figure 9. Due to the shear lag effect of the GFRP composite materials, their tensile failure mode exhibited a lantern-like pattern.

In the pullout test, regardless of the strengths of the concrete blocks, all seven GFRP bolts experienced rod fracture, as shown in Figure 8 and Figure 9. The ultimate tensile strengths of the GFRP bolts embedded in the three concrete blocks were recorded as 1330.56, 1319.62, and 1308.67 MPa, with an average value of 1319.62 MPa. Typically, it is recommended to take 75% of the ultimate tensile strength value of GFRP rods as the design value in practical engineering. Due to the high shear strength between the anchor agent and the rod, as well as between the anchor agent and the concrete, the experimental results consistently indicated rod fracture, regardless of the strength of the simulated rock concrete blocks. This observation differed from the bonding strength calculation formulas endorsed by the American Concrete Institute (Equation (2)) for reinforced concrete structures [15] and those proposed by Okelo et al. [14] in their scholarly works (Equation (3)):

(2) $$u_{c}=20.23\frac{\sqrt{f_{c}^{\prime }}}{d_{b}}\;(\mathrm{MPa}),$$

(3) $$u_{c\_FRP}=14.70\frac{\sqrt{f_{c}^{\prime }}}{d_{b}}\;(\mathrm{MPa}),$$

where $f_{c}^{\prime }$ is the compressive strength of concrete (MPa), and *d_b_* is the rod diameter (mm).

**Figure 10:**
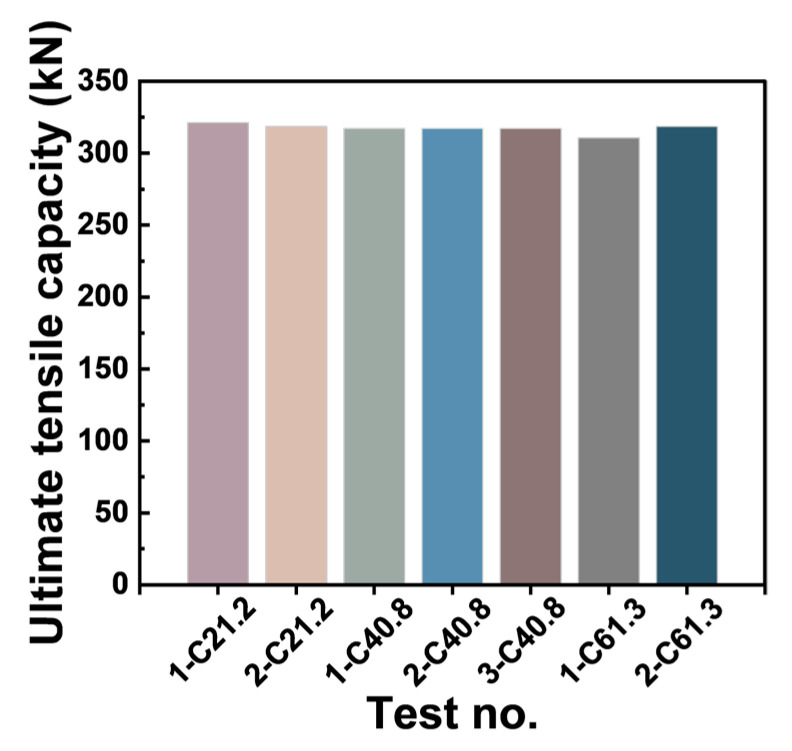
Ultimate tensile capacity of the GFRP bolts.

In this GFRP anchoring system, the pullout tests consistently resulted in rod fracture failure. Consequently, the interfacial bonding strengths of the GFRP bolts are not controlled via the compressive strengths of the concrete blocks. The tensile capacity of GFRP bars could be fully utilized (Figure 10). By contrast, steel-reinforced concrete or FRP-reinforced concrete structures typically exhibited interfacial slip failure modes, characterized by direct rod-to-concrete contact and the absence of grouting materials.

## 4. Conclusions

In marine protection projects, the durability of steel anchor structures with traditional cement-based materials as grouting materials is poor. In order to address this issue, a new solution was proposed: epoxy resin was used as an anchoring agent, GFRP bars were used instead of steel bars, and a large concrete platform was used to simulate rock mass. The durability of GFRP resin bolts and the pullout performance of GFRP resin anchors in large-scale concrete blocks were experimentally studied in this work. According to the above results and analysis, the following conclusions were drawn.

(1) The GFRP rods and epoxy resin exhibited excellent corrosion resistance. The strength variations of the GFRP rods and epoxy resin materials in artificial seawater environments were less than 5%. Therefore, the GFRP resin bolts were suitable for coastal protection engineering. The stress–strain relationship of GFRP tendon is approximately linear with no yield point, and its failure form is a brittle failure. It is recommended to take 75% of the ultimate tensile strength as the design value. Compared with carbon steel, GFRP has the advantages of an outstanding high strength/stiffness-to-weight ratio, ease of transportation, cutting, and installation. However, its modulus of elasticity is relatively low and should be a concern in practical engineering design.
(2) Laboratory test results show that, when the bolt diameter and resin strength were constant, the pull load on the GFRP bolts embedded in the steel pipe along the anchoring length gradually increased and then stabilized with the increase in the anchorage length. A critical value was observed for the anchorage length of the GFRP bolts.
(3) Due to the high shear strength between the resin and the rod, as well as between the resin and the concrete, the results of the pullout tests for the seven GFRP resin bolts embedded in the three types of concrete blocks with different compressive strengths all exhibited rod fracture failure. The results showed that the failure mode was not controlled via the compressive strength of concrete blocks. The GFRP resin anchor system could fully utilize the tensile capacity of the GFRP rods. It can provide reference and guidance for practical engineering applications.

## Figures and Tables

**Table 1:** Mix proportions of corrosion solution (Unit: g).

Number	Water:Salt:Sulfuric Acid	pH
1	100:0:0	7.20
2	100:15:0	9.34
3	100:30:0	9.86
4	100:15:15	0.57
5	100:0:15	0.55
6	100:0:30	0.19

**Table 2:** Physical and mechanical properties of the full-threaded GFRP bars.

Nominal Diameter (mm)	Tensile Strength (MPa)	Torque (N·m)	Shear Strength (MPa)	Area (mm^2^)	Weight (kg/m)
22	900	90	140	240.4	0.683

**Table 3:** Strength indices of the modified epoxy resin materials.

Compressive Strength (MPa)	Flexural Strength (MPa)	Splitting Tensile Strength (MPa)	Bond Strength (MPa)
57.4	21.8	10.1	5.6

**Table 4:** Configuration of the corrosive solutions and test specimens.

Corrosive Solution	Water:Salt:Sulfuric Acid (by Weight)	Number of GFRP Bars	Anchoring Agent Block
1	100:0:0	3	-
2	100:15:0	-	15
3	100:30:0	3	15
4	100:15:15	3	15
5	100:0:15	-	15
6	100:0:30	3	15
Total	-	12	75

**Table 5:** Mechanical indicators of GFRP rods after 150 days of corrosion.

Corrosive Solution	Water:Salt:Sulfuric Acid (by Weight)	Tensile Strength (MPa)	Elastic Modulus (MPa)	Elongation Rate (%)
1	100:0:0	1139.00	4.48 × 10^4^	2.14
3	100:30:00	1102.51	4.33 × 10^4^	2.12
4	100:15:15	1120.76	4.41 × 10^4^	2.29
6	100:00:30	1138.09	4.37 × 10^4^	2.11

**Table 6:** Compressive strengths of the simulated rock mass concrete.

Specimen Number	Pressure (ton)	Specimen Size	Compressive Strength (MPa)	Average Compressive Strength (MPa)
C20-1	48	150 mm × 150 mm × 150 mm	21.3	21.2
C20-2	51		22.6	
C20-3	50		22.2	
C20-4	46		20.4	
C20-5	45		20.0	
C20-6	47		20.9	
C40-1	91		40.4	40.8
C40-2	93		41.3	
C40-3	92		40.8	
C60-1	140		62.2	61.3
C60-2	136		60.4	
C60-3	138		61.3	

**Table 7:** Pullout test results of the GFRP bolts with different anchor lengths.

Diameter (mm)	Anchor Length (cm)	Ultimate Pull Load (kN)	Failure Mode
22	10	150.13	Slip between the rod and resin
22	15	271.18	Rod fracture
22	20	263.29	Rod fracture
22	30	271.18	Rod fracture

**Table 8:** Pullout test results of the GFRP bolts in concrete with different strengths.

Anchor Number	Ultimate Pullout Force (KN)	Ultimate Tensile Strength (MPa)	Average Ultimate Tensile Strength (MPa)
1-C21.2	321.18	1336.04	1330.56
2-C21.2	318.55	1325.09	
1-C40.8	317.24	1319.62	1319.62
2-C40.8	317.24	1319.62	
3-C40.8	317.24	1319.62	
1-C61.3	310.66	1292.25	1308.67
2-C61.3	318.55	1325.09	

## Data Availability

The data used to support the findings of this study (including the performance evaluation of the epoxy resin during the corrosion process) are available in the Supplementary Information.

## Supplementary Materials

The following supporting information can be downloaded at: https://www.mdpi.com/article/10.3390/ma17122814/s1, Figure S1: Changes in weight of the epoxy resin materials with corrosion time; Figure S2: The variation of compressive strength of epoxy resin with corrosion time under five corrosive solutions; Table S1: Changes in weight of the epoxy resin materials with corrosion time.
