# Supplementary material for: Experimental Study on Durability and Bond Properties of GFRP Resin Bolts
Source: Materials (Basel). 2024 Jun 9;17(12):2814. doi: 10.3390/ma17122814 (PMC11204529; doi:10.3390/ma17122814)
Supplement: Supplementary file 1 [file materials-17-02814-s001.zip › materials-3001861-supplementary.pdf]

Materials

Supplementary Information for

Experimental Study on Durability and Bond Properties of GFRP Resin Bolts

Mingan Lin [1], Fuming Zhang [2] and Wei Wang [3,*]

[1] School of Civil Engineering, Southwest Jiaotong University, Chengdu 610031, China; change21c@163.com
[2] Institute of Defense Engineering, Academy of Military Sciences (AMS), People's Liberation Army (PLA), Beijing 100036, China; dzj0379@163.com
[3] Key Laboratory of Impact and Safety Engineering, Ningbo University, Ministry of Education, Ningbo 315211, China
* Correspondence: wangwei7@nbu.edu.cn

Contents of this file

Text S1 to S2
Table S1
Figures S1 to S2

Introduction

This file includes two supporting text (Text S1 to S2), one supporting table (Table S1)and two supporting figures (Figures S1 to S2). Text S1 is the evaluation of the changes in weight of the epoxy resin materials before and after corrosion, and the detailed experiment data is shown in Table S1 and Figure S1. Text S2 gives detailed data about the variations in compressive strength of the epoxy resin materials in five different corrosive solutions. Figure S2 shows the curve depicting the change in compressive strength of this material during the corrosion process in this study.

Text S1: Evaluation of the changes in weight of the epoxy resin materials before and after corrosion

To quantitatively evaluate the changes in weight of the epoxy resin materials with corrosion time, we compared and analyzed the performance of the epoxy resin anchoring adhesive specimens before and after Corrosion. Table S1 presents the variations in weight of the epoxy resin materials in 5 different corrosive solutions over time, and Figure S1 presents the curves depicting the change in weight of this material during the corrosion process. We observed that the weight of the epoxy resin material experienced minimal change with increasing corrosion time.Notably, after 126 days of corrosion in the 100:0:30 strong acid

solution, the weight increased by 1.7%, while in the other corrosive solutions, the weight decreased by 0.4% to 1.3%, which was considered negligible.

Corrosive Solution (Water:Salt:Sulfuric Acid)	Weight Before Corrosion (g)	Weight After 7 Days (g)	Weight After 31 Days (g)	Weight After 62 Days (g)	Weight After 90 Days (g)	Weight After 126 Days (g)
100:15:00	95.97	95.93	94.87	95	94.77	95.57
100:30:00	95.97	98.03	96.10	95.87	94.83	95.17
100:15:15	95.97	93.53	96.20	95.23	94.90	94.70
100:00:15	95.97	95.10	95.53	94.97	95.40	94.93
100:00:30	95.97	94.93	98.80	95.27	96.80	97.60

Table S1. Changes in weight of the epoxy resin materials with corrosion time.

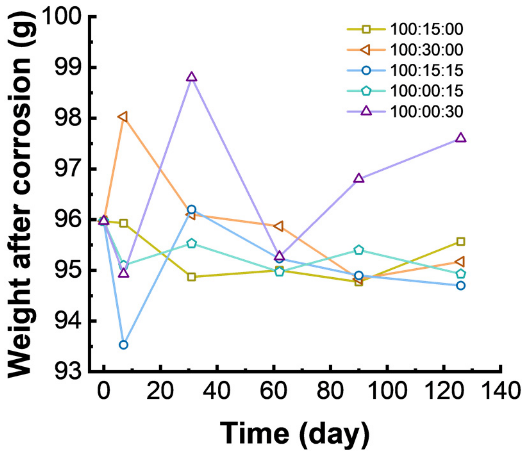

Figure S1. Changes in weight of the epoxy resin materials with corrosion time.

Text S2: Evaluation of the variations in compressive strength of the epoxy resin materials during the corrosion process.

Figure S2 shows the the change in compressive strength of the epoxy resin material during the corrosion process in this study. The change in compressive strength is slightly significant, demonstrating a substantial linear increase with corrosion time (Figure S2). Notably, in the 126-day salt corrosion environment, the average increase in compressive strength was 2.6%, while in the 126-day salt and acid corrosion environment, the average increase in compressive strength was 2.8%. In the 126-day acid corrosion environment, the average increase in compressive strength was 2.7%. In general, the strength variations of the epoxy resin materials in harsh corrosive environments were less than 5%.

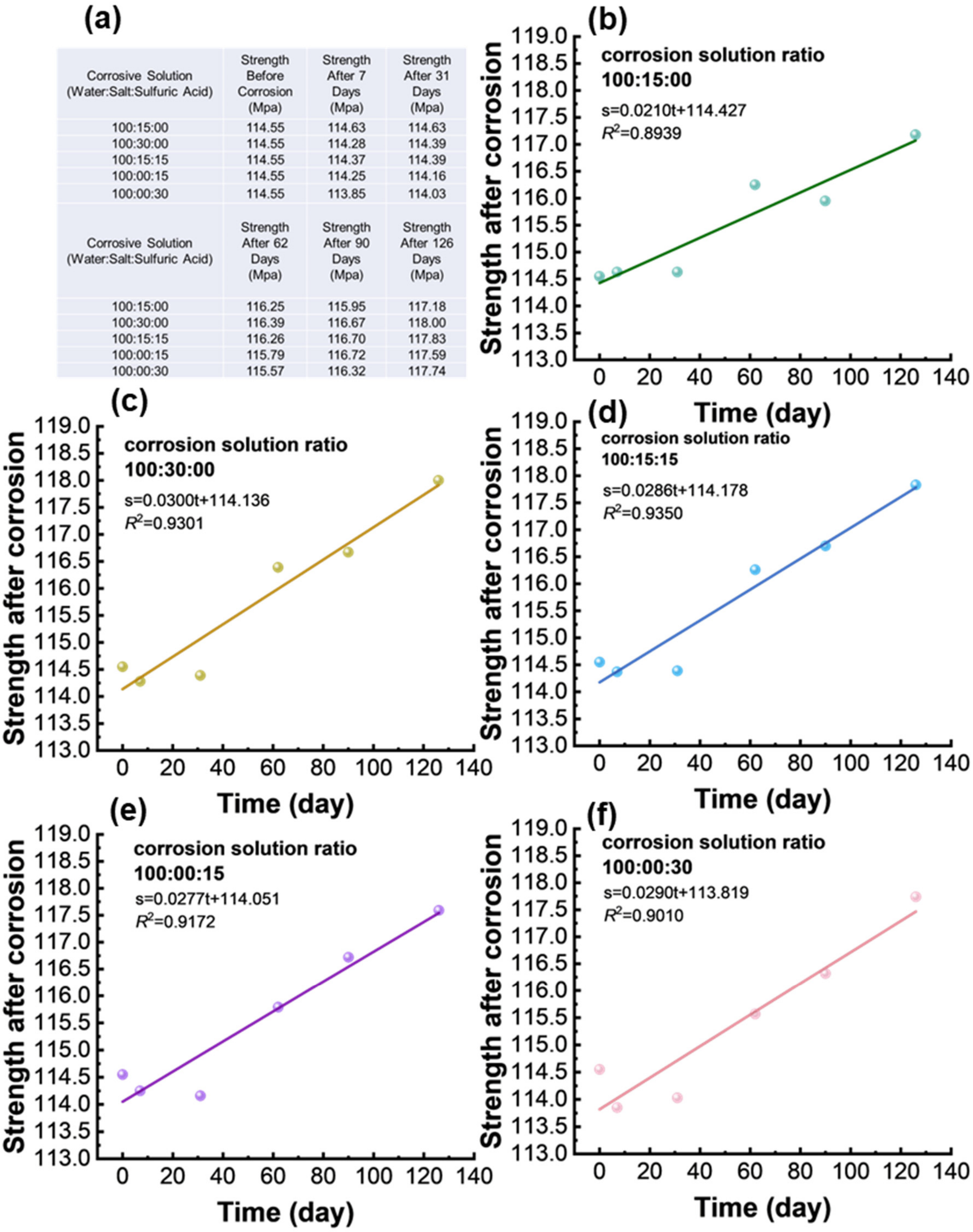

(a)

Corrosive Solution (Water:Salt:Sulfuric Acid)	Strength Before Corrosion (Mpa)	Strength After 7 Days (Mpa)	Strength After 31 Days (Mpa)
100:15:00	114.55	114.63	114.63
100:30:00	114.55	114.28	114.39
100:15:15	114.55	114.37	114.39
100:00:15	114.55	114.25	114.16
100:00:30	114.55	113.85	114.03

Corrosive Solution (Water:Salt:Sulfuric Acid)	Strength After 62 Days (Mpa)	Strength After 90 Days (Mpa)	Strength After 126 Days (Mpa)
100:15:00	116.25	115.95	117.18
100:30:00	116.39	116.67	118.00
100:15:15	116.26	116.70	117.83
100:00:15	115.79	116.72	117.59
100:00:30	115.57	116.32	117.74

Figure S2. (a) The data of the variation of compressive strength of epoxy resin with corrosion time under five different corrosion solution ratios; **(b)-(f)** The fitted curves of the variation of compressive strength of epoxy resin with corrosion time under five different corrosion solution ratios.
